# Calycosin-7-*O*-*β*-*D*-glucoside Attenuates OGD/R-Induced Damage by Preventing Oxidative Stress and Neuronal Apoptosis via the SIRT1/FOXO1/PGC-1*α* Pathway in HT22 Cells

**DOI:** 10.1155/2019/8798069

**Published:** 2019-12-01

**Authors:** Xiangli Yan, Aiming Yu, Haozhen Zheng, Shengxin Wang, Yingying He, Lisheng Wang

**Affiliations:** College of Chinese Materia Medica, Guangzhou University of Chinese Medicine, Guangzhou, 51006 Guangdong, China

## Abstract

Neuronal apoptosis induced by oxidative stress is a major pathological process that occurs after cerebral ischemia-reperfusion. Calycosin-7-*O*-*β*-*D*-glucoside (CG) is a representative component of isoflavones in *Radix Astragali* (RA). Previous studies have shown that CG has potential neuroprotective effects. However, whether CG alleviates neuronal apoptosis through antioxidant stress after ischemia-reperfusion remains unknown. To investigate the positive effects of CG on oxidative stress and apoptosis of neurons, we simulated the ischemia-reperfusion process *in vitro* using an immortalized hippocampal neuron cell line (HT22) and oxygen-glucose deprivation/reperfusion (OGD/R) model. CG significantly improved cell viability and reduced oxidative stress and neuronal apoptosis. In addition, CG treatment upregulated the expression of SIRT1, FOXO1, PGC-1*α*, and Bcl-2 and downregulated the expression of Bax. In summary, our findings indicate that CG alleviates OGD/R-induced damage via the SIRT1/FOXO1/PGC-1*α* signaling pathway. Thus, CG maybe a promising therapeutic candidate for brain injury associated with ischemic stroke.

## 1. Introduction

Ischemic stroke is a neurodegenerative disease characterized by hypoxemia of the brain tissue due to vascular obstruction. This condition is characterized by high morbidity, disability, mortality, and high recurrence rate, thus creating a heavy burden on society [1–3]. When the blood supply is blocked, many pathological mechanisms contribute to cell death, including oxidative stress, inflammation, glutamate and calcium toxicity, and mitochondrial dysfunction [4]. The tissue plasminogen activator (tPA) and plasminogen activator inhibitor 1 (PAI-1) are the key players of the fibrinolytic plasminogen activator system. The role of PAI-1 in brain injury has been established [5, 6]. tPA is the only drug approved by the US FDA for treating ischemic stroke [7]. However, this drug is hampered by its narrow therapeutic window and can cause secondary damage to the ischemic area, known as ischemia-reperfusion injury [8–10]. It is estimated that only 5-7% of ischemic stroke patients receive tPA intravenous injection [11, 12]. Therefore, it is imperative to find new and effective drugs for treating ischemic stroke.

**Figure 1:**
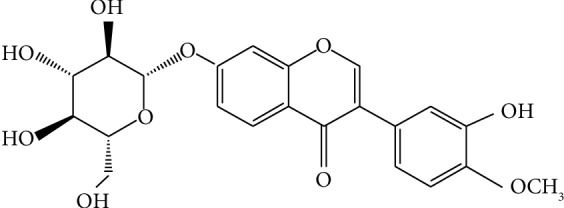
Structure of Calycosin-7-*O*-*β*-*D*-glucoside.

*Radix Astragali* (RA), also known as Huangqi in China, is the dried root of *Astragalus membranaceus* [13]. Previous bioactive studies showed that several types of bioactive components in RA, such as isoflavonoids, triterpene saponins, and polysaccharides, have a wide variety of biological activities such as cardioprotection, anti-inflammation, and antioxidative stress effects [14–18]. Calycosin-7-*O*-*β*-*D*-glucoside (CG, Figure 1) is a representative isoflavone isolated from RA, and studies have shown that CG may have neuroprotective effects [17]. CG was shown to attenuate ischemia-reperfusion injury by activating the PI3K/Akt pathway [19] and reducing myocardial injury in heat stroke rats through its anti-inflammation effects [20]. However, the underlying mechanism remains unclear.

Silent information regulator 1 (SIRT1) is a histone deacetylase, and its activity is mainly dependent on nicotinamide adenine dinucleotide (NAD^+^) [21]. It plays a critical role in protecting against ischemic stroke [22]. In addition, it is considered as a lifespan gene because it plays an important role in regulating longevity [23, 24]. Forkhead box O (FOXO) and peroxisome proliferator-activated receptor *γ* coactivator-1 (PGC-1*α*) may be the direct substrates of SIRT1 [25]. Numerous studies have shown that when SIRT1 is activated, it can exert antioxidative stress, antiapoptosis, and anti-inflammatory effects by deacetylating downstream FOXO1 and PGC-1*α* [26–29]. Interestingly, previous studies showed that CG can alleviate the damage caused by ischemic stroke [13]. Given the protective effects of CG on ischemic stroke, we hypothesized that CG protects against ischemic stroke via the SIRT1/FOXO1/PGC-1*α* signaling pathway.

To investigate the positive effects of CG on apoptosis induced by ischemic stroke, we used the immortalized mouse hippocampal neuron cell line (HT22) and oxygen glucose deprivation reperfusion (OGD/R) model to mimic ischemic reperfusion *in vitro*.

## 2. Materials and Methods

### 2.1. Reagents

CG was from Chengdu Keloma Biotechnology Co., Ltd. (Chengdu, China); the purity was greater than 98%. Nimodipine was purchased from Guangdong Huanan Pharmaceutical Group Co., Ltd. (Guangdong, China). Dulbecco's modified Eagle's medium (DMEM), glucose-free DMEM, fetal bovine serum (FBS), and penicillin-streptomycin were obtained from Gibco (Grand Island, NY, USA). Cell Counting Kit-8 (CCK-8) was obtained from Dojindo Laboratories (Kumamoto, Japan). Lactate dehydrogenase (LDH), superoxide dismutase (SOD), and malondialdehyde (MDA) assay kits were obtained from Nanjing Jiancheng Bioengineering Institute (Nanjing, China). The Annexin V-FITC Apoptosis Detection Kit was from KeyGEN BioTECH (Nanjing, China). The reactive oxygen species (ROS) Detection Kit was obtained from BestBio (Shanghai, China). PGC-1*α* enzyme-linked immunosorbent assay (ELISA) kits were from Jiangsu Enzyme Biotechnology Co., Ltd. (Jiangsu, China). Anti-SIRT1 antibody was supplied by Cell Signaling Technology (Danvers, MA, USA). Anti-FOXO1, anti-Bcl-2, anti-Bax, and anti-*β*-actin antibodies, and goat anti-rabbit secondary antibody were purchased from Affinity (Cincinnati, OH, USA). The TRIzol reagent was supplied by Takara Biomedical Technology (Shiga, Japan). The reverse-transcribed cDNA synthesis kit and qPCR kit were from Beijing TsingKe Biotech Co., Ltd. (Beijing, China).

### 2.2. OGD/R Model and Medical Treatment

HT22 cells were cultured in medium composed of DMEM, 10% FBS, 100 U/mL penicillin, and 100 mg/mL streptomycin in the cell culture incubator at 37°C under 5% CO_2_. When the cell density reached 70–80%, the medium was removed and the cells were washed three times with phosphate-buffered saline, followed by incubation in a three-gas incubator (94% N_2_, 5% CO_2_, and 1% O_2_) with glucose-free medium for 8 h at 37°C to mimic hypoxic injury (OGD). Next, the medium was replaced with normal medium, and the cells were incubated in an incubator with 5% CO_2_ at 37°C for another 6 h. In addition to reperfusion treatment, the cells were treated with CG (15 *μ*g/mL) or nimodipine (2.5 *μ*g/mL), a drug used clinically to treat ischemic brain damage. Cells cultured in complete medium and normal environment were used as the control group.

### 2.3. Cell Viability

Cell viability was detected by the CCK-8 assay. Briefly, cells were seeded in 96-well plates at a density of 1.5 × 10^4^ cells/cm^2^; after treatment, CCK-8 was added to the medium for another 2 h at 37°C. Absorbance was measured at 450 nm using a microplate reader. Cell viability was shown as a percentage of the control group. The experiment was repeated three times.

### 2.4. LDH Release Assay

The release of LDH represents the integrity of the cell membrane [30]. LDH activity was measured with an LDH assay kit according to the manufacturer's instructions. Cells were seeded in 96-well plates; after exposure to OGD/R, the cells were collected and sonicated, and then centrifuged to collect the supernatant for analysis. The supernatant was reacted with substrate solution at 37°C for 15 min. It was then reacted with 2,4-dinitrophenylhydrazine for another 15 min. The absorbance was detected with a microplate reader at 450 nm. The results were expressed as a multiple relative to the control group. The experiment was performed in triplicate.

### 2.5. MDA Content and SOD Activity

Cells were seeded in 96-well plates; after treatment, the cells were sonicated and centrifuged to collect the supernatant. The thiobarbituric acid method was used to detect the MDA content. The supernatant was reacted with WST (a highly water-soluble tetrazolium salt) and enzyme working solution to measure SOD activity. All operations were according to the manufacturer's instructions. The results were shown as a multiple relative to the control group. The experiment was replicated thrice.

### 2.6. Detection of ROS

The levels of ROS in hippocampal cells were assessed with the fluorescent probe 2′,7′-dichlorfluorescein-diacetate (DCFH-DA) kit. Briefly, cells were seeded in 96-well plates; after exposure to OGD/R, the cells were reacted with the working solution for 20 min at 37°C in the dark. After washing 3 times with serum-free medium, images were obtained under a fluorescence microscope. The experiment was repeated three times.

### 2.7. Cell Apoptosis Assay

Apoptosis was detected by using an Annexin V-FITC Apoptosis Detection Kit. Cells were seeded in 10 cm cell culture dishes at a density of 1.5 × 10^4^ cells/cm^2^, according to the supplier's instructions; after treatment, the cells were collected by adding trypsin without EDTA, followed by washing twice with phosphate-buffered saline and centrifugation for 5 min at 2000 rpm. The cells were suspended in binding buffer and incubated with FITC-labeled Annexin V and propidium iodide (PI) at room temperature in the dark for 10 min. The apoptotic rate was measured by flow cytometry within 1 h. The experiment was performed in triplicate.

### 2.8. PGC-1*α* Content

The expression of PGC-1*α* was detected with an ELISA kit according to the manufacturer's instructions. Briefly, cells were seeded in 96-well plates; after exposure to OGD/R, the cells were lysed and centrifuged. The supernatant was added to the bottom of the plate and incubated at 37°C for 30 min. After washing 5 times, the enzyme labeling reagent was added and incubated for 30 min, followed by washing and addition of color developer which was incubated with the sample for 10 min. The absorbance was detected with a microplate reader at 450 nm. The experiment was replicated thrice.

### 2.9. Western Blotting

Cells were seeded in 10 cm cell culture dishes; after treatment, total protein was extracted from cells lysed by RIPA, and the protein concentration was determined with a BCA protein assay kit. Ten *μg* of total protein samples was loaded into an 8–10% polyacrylamide gel for separation by SDS-PAGE and then transferred to polyvinylidene fluoride membranes. After the membranes were blocked with 5% nonfat milk, they were incubated with primary antibodies against SIRT1 (1 : 1000), FOXO1 (1 : 1000), Bcl-2 (1 : 1000), Bax (1 : 1000), and *β*-actin (1 : 1000) at 4°C overnight. The membrane was incubated with secondary antibodies (1 : 3000) for 1 h at room temperature. Finally, the membranes were exposed using the Bio-Rad ChemiDoc Touch Imaging System (Bio-Rad, Hercules, CA, USA) and analyzed with ImageJ software (NIH, Bethesda, MD, USA). The relative protein levels were normalized to that of *β*-actin. The experiment was repeated three times.

### 2.10. Real-Time Polymerase Chain Reaction (PCR)

Cells were seeded in 10 cm cell culture dishes; after exposure to OGD/R, total RNA was extracted from hippocampal cells after OGD/R by using TRIzol reagent and one *μg* of RNA was reverse transcribed to cDNA using the reverse-transcribed cDNA synthesis kit. Real-time PCR was performed on an ABI 7500 Sequence Detection System (Applied Biosystems, Foster City, CA, USA). The sequences of primers were as follows: SIRT1: forward: 5′-CTCCTTGGAGACTGCGATGT-3′, reverse: 5′-GTGTTGGTGGCAACTCTGAT-3′; FOXO1: forward: 5′-AAGTACACATACGGCCAATCC-3′, reverse: 5′-GGGAGGAGAGTCAGAAGTCA-3′; PGC-1*α*: forward: 5′-AAGGTCCCCAGGCAGTAGAT-3′, reverse: 5′-TCCCTCTTGAGCCTTTCGT-3′; Bcl-2: forward: 5′-AGCCTTGGCCAGGGAATTAT-3′, reverse: 5′-GGACTTGGTGCATGGAACAC-3′; Bax: forward: 5′-GAACTGGACAGCAATATGGA-3′, reverse: 5′-GAAGTTGCCATCAGCAAAC-3′; and *β*-actin: forward: 5′-GCTTCTAGGCGGACTGTTAC-3′, reverse: 5′-CCATGCCAATGTTGTCTCTT-3′. All operations were repeated three times, and the data were indicated with 2^-*ΔΔ*Ct^ method and expressed as fold difference normalized to *β*-actin.

### 2.11. Statistical Analysis

Results were presented as the mean ± SD from three independent experiments. The significance of differences between two groups was analyzed by Student's *t*-test, while the significance of differences between more than two groups was assessed by one-way analysis of variance (ANOVA). SPSS 17.0 software (SPSS, Inc., Chicago, IL, USA) was used for the analyses. A value of *P* < 0.05 was considered as statistically significant.

## 3. Results

### 3.1. CG Protects Hippocampal Cells against OGD/R-Induced Injury

**Figure 2:**
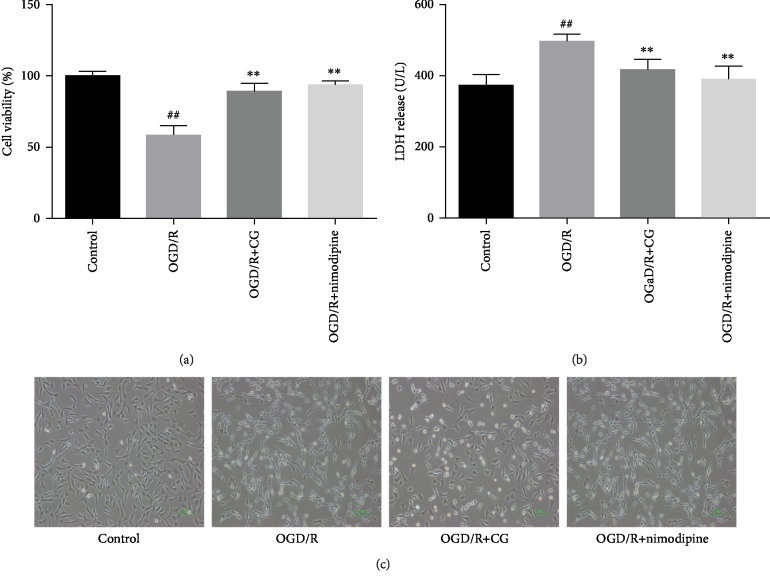
HT22 cells were treated with 8 h of OGD and then reoxygenated in the presence of CG for 6 h. (a) Cell viability was detected by CCK-8 assay. (b) Cytotoxicity was determined with LDH assays. (c) Cell morphology was evaluated by a biological microscope. Three independent experiments were performed, and data were expressed as the mean ± SD. ^##^*P* < 0.01*vs.* control group, ^∗∗^*P* < 0.01*vs.* OGD/R group.

To explore the protective effects of CG on hippocampal cells after OGD/R, we first tested the cell viability and release of LDH. As shown in Figures 2(a) and 2(b), after the cells were treated with OGD/R, the viability of cells reduced to 64% and the release of LDH increased to 505 U/L (*P* < 0.01), indicating that the OGD/R model was successfully established. However, CG alleviated cell death and LDH leakage caused by OGD/R. Additionally, OGD/R injury caused cell shrinkage, but after CG intervention, the cell morphology tended to be normal (Figure 2(c)). These results indicate that CG alleviates OGD/R-induced cell injury.

### 3.2. CG Alleviates OGD/R-Induced Oxidative Stress in Hippocampal Cells

**Figure 3:**
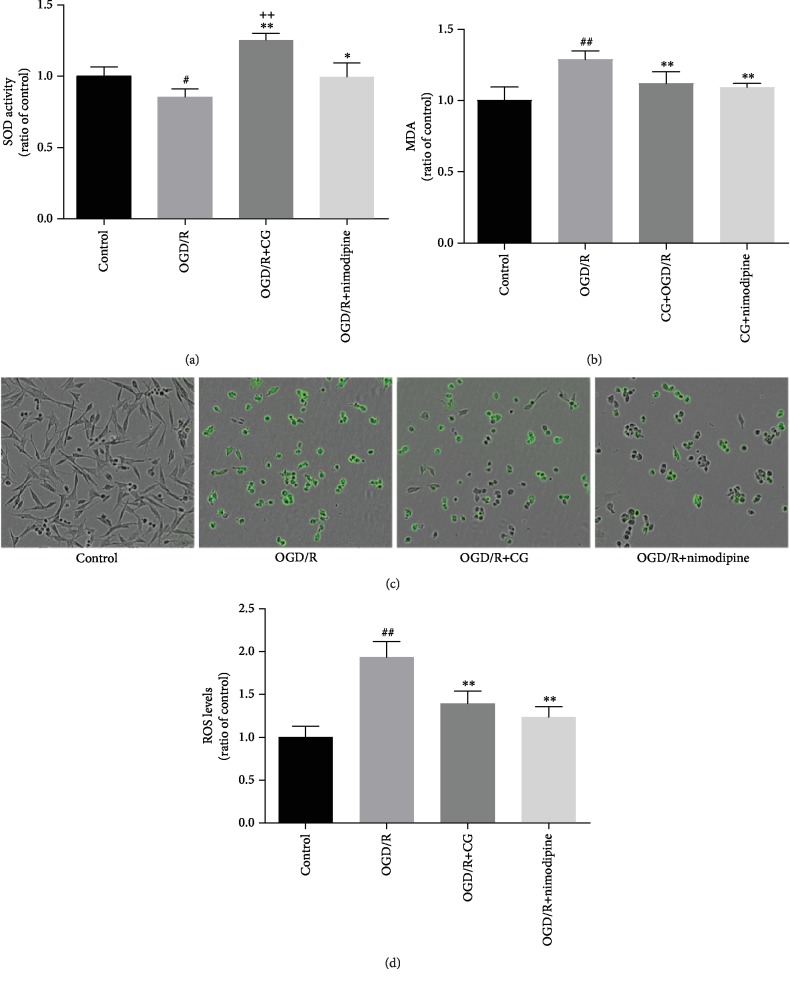
HT22 cells were treated with 8 h of OGD and then reoxygenated in the presence of CG for 6 h. CG elevated the SOD activity and reduced the MDA level and ROS generation: (a) SOD activity; (b) MDA level; (c) ROS fluorescence intensity; (d) ROS level. Three independent experiments were performed, and data were expressed as the mean ± SD. ^#^*P* < 0.05, ^##^*P* < 0.01*vs.* control group; ^∗^*P* < 0.05, ^∗∗^*P* < 0.01*vs.* OGD/R group; ^++^*P* < 0.01*vs.* nimodipine group.

Oxidative stress plays a major role in ischemia-reperfusion injury [31, 32]. As shown in Figures 3(a)–3(c), we observed a significant increase in MDA and ROS levels and decrease in SOD activity compared to those in the normal group. However, the changes were reversed by CG administration. These results indicate that CG significantly affects oxidative damage caused by OGD/R.

### 3.3. CG Alleviates OGD/R-Induced Apoptosis

**Figure 4:**
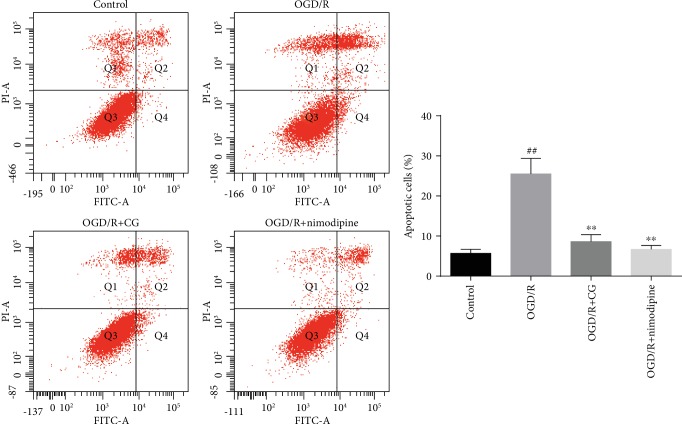
Apoptosis rate of HT22 cells after treatment with 8 h of OGD and reoxygenated in the presence of CG for 6 h. Three independent experiments were performed, and data were expressed as the mean ± SD. ^##^*P* < 0.01*vs.* control group, ^∗∗^*P* < 0.01*vs.* OGD/R group.

The hippocampus is very vulnerable to ischemia, and ischemia-reperfusion leads to neuronal apoptosis [33, 34]. To further investigate the protective effect of CG on OGD/R hippocampal cells, we examined the apoptosis rate. As shown in Figure 4, the apoptosis rate increased from 5-28% after exposure to OGD/R (*P* < 0.01), whereas treatment with CG alleviated apoptosis caused by OGD/R. Taken together, these results demonstrate that CG can alleviate OGD/R-induced apoptosis.

### 3.4. CG Regulates SIRT1/FOXO1/PGC-1*α* Signaling Pathways in Hippocampal Cells

**Figure 5:**
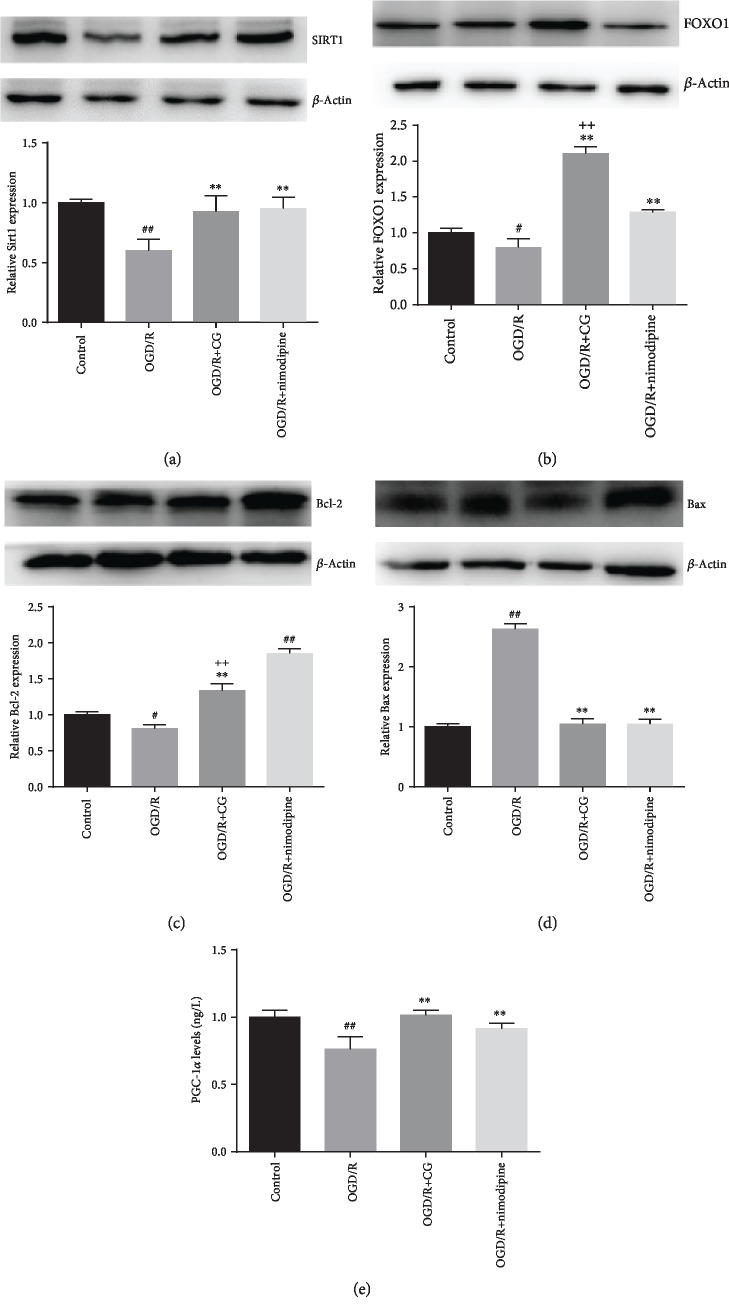
HT22 cells were treated with 8 h of OGD and then reoxygenated in the presence of CG for 6 h. Protein expression of SIRT1 (a), FOXO1 (b), Bcl-2 (c), and Bax (d) detected by Western blotting and PGC-1*α* (e) detected by ELISA. Three independent experiments were performed, and data were expressed as the mean ± SD. ^#^*P* < 0.05, ^##^*P* < 0.01*vs.* control group; ^∗∗^*P* < 0.01*vs.* OGD/R group; ^++^*P* < 0.01*vs.* nimodipine group.

**Figure 6:**
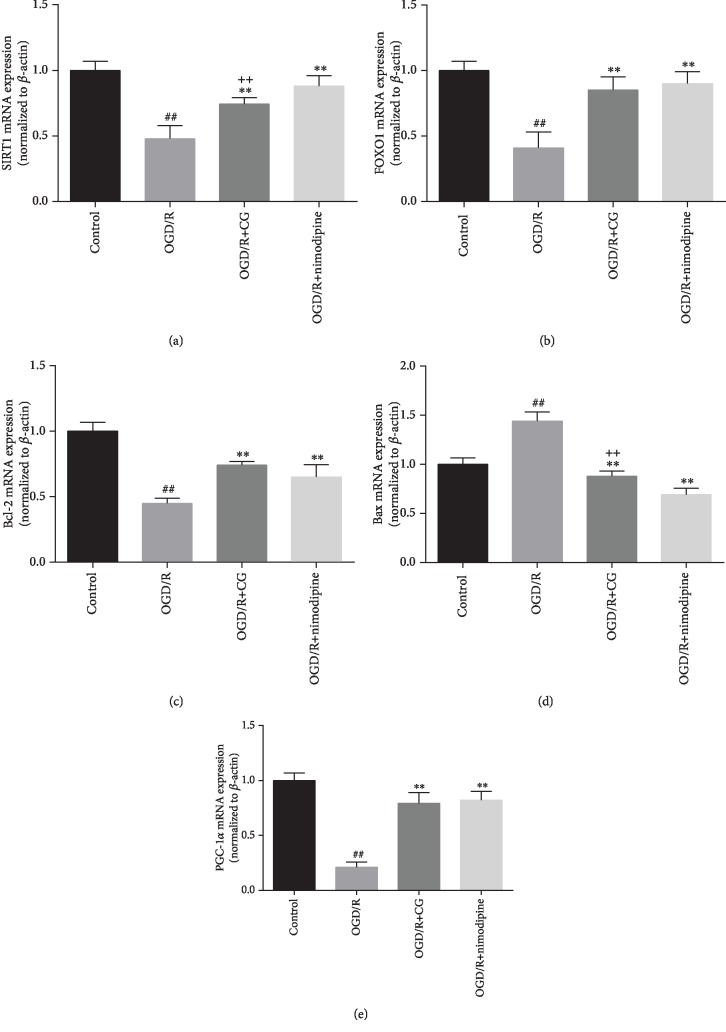
The expression of SIRT1 (a), FOXO1 (b), Bcl-2 (c), Bax (d), and PGC-1*α* (e) mRNA after HT22 cells were treated with 8 h of OGD and reoxygenated in the presence of CG for 6 h. Three independent experiments were performed, and data were expressed as the mean ± SD. ^##^*P* < 0.01*vs.* control group; ^∗∗^*P* < 0.01*vs.* OGD/R group; ^++^*P* < 0.01*vs.* nimodipine group.

To investigate the mechanism of CG in hippocampal neuronal cells after OGD/R, we evaluated the effects of CG on the SIRT1/FOXO1/PGC-1*α* signaling pathway related to apoptosis. As shown in Figure 5, after OGD/R, the protein expression of SIRT1, FOXO1, PGC-1*α*, and Bcl-2 was significantly reduced. However, CG treatment reversed these inhibitions. Additionally, the protein expression of Bax was increased after OGD/R compared to that in the control group, whereas CG downregulated the expression of Bax. We also examined the effects of CG intervention on the mRNA expression of SIRT1, FOXO1, PGC-1*α*, Bcl-2, and Bax (Figure 6), and the results were consistent with those of Western blotting and ELISA. These results indicate that CG can alleviate damage to OGD/R to hippocampal neurons via the SIRT1/FOXO1/PGC-1*α* signaling pathway.

## 4. Discussion

Despite tremendous advances in the theoretical understanding of ischemic stroke in the past several decades, stroke remains the third leading cause of death and permanent disability [35]. Oxygen glucose deprivation reperfusion is an *in vitro* model that mimics the *in vivo* process of a series of pathological reactions initiated by ischemia-reperfusion [36, 37]. Resveratrol is a natural polyphenol compound [38]. It has significant protective effects on ischemic stroke, such as antiapoptotic, antioxidant, anti-inflammatory, and neuroprotective properties [39–41]. In addition, it can promote synaptogenesis and neurite outgrowth and prevents axonal degeneration after injury [42, 43]. However, there are few natural active ingredients which have similar efficacy to resveratrol. CG has been reported to protect cardiomyocytes from ischemia/reperfusion injury [19] and endothelial cells from bacterial endotoxin-induced vascular cell injury [16]. Liu et al. found six core bioactive components in Xueshuantong capsule to promote blood circulation, among which CG showed obvious anticoagulant activity [44]. Some studies have shown that CG could eliminate oxygen-free radicals [45, 46] and inhibit high glucose-induced mesangial cell proliferation and glomerular endothelial cell apoptosis [47]. However, its exact role is unclear. In this study, we demonstrated that CG prevented OGD/R-induced hippocampal cell injury by alleviating oxidative stress and apoptosis through the SIRT1/FOXO1/PGC-1*α* signaling pathway.

Cerebral ischemia-reperfusion leads to imbalanced ROS production and clearance, followed by oxidative stress [48]. Accumulating evidence has indicated that oxidative stress plays a major role in OGD/R and is directly related to the clinical prognosis of ischemic stroke [49, 50]. A recent study confirmed the antioxidative effects of CG [16]. Consistent with previous findings, our data indicated that CG can reduce OGD/R-induced ROS and MDA production and decrease SOD activity, suggesting that CG attenuates OGD/R-induced hippocampal neuronal damage through antioxidative stress and by inhibiting apoptosis.

SIRT1 senses changes in the cellular environment through the redox state of NAD^+^/NADH and participates in antiapoptosis, anti-inflammatory, and metabolism processes to enhance cell viability by deacetylation by FOXO1 and PGC-1*α* [51–55]. The FOXO family is closely related to apoptosis and oxidative stress. Studies have shown that SIRT1 can exert antioxidative stress by regulating the PTEN/JNK/FOXO1 signaling pathway [56]. PGC-1*α* plays an important role in neuroprotection [57]. It has been reported that SIRT1 promotes the transcription of PGC-1*α* to regulate the expression of Bcl-2 and Bax proteins, thereby increasing the antioxidant capacity of nerve cells [58]. In the present study, we found that CG treatment upregulated the expression of SIRT1, FOXO1, and PGC-1*α*. Additionally, the expression of Bcl-2 and Bax was analyzed. The results indicated that CG can increase Bcl-2 and decrease Bax expression. The results showed that CG reduces oxidative stress and neuronal apoptosis through the SIRT1/FOXO1/PGC-1*α* pathway.

## 5. Conclusions

In conclusion, our results suggest that CG protects against ischemic stroke by activating SIRT1, which in turn upregulates FOXO1 and PGC-1*α* expression. Taken together, these findings indicate that CG alleviates OGD/R-induced damage via the SIRT1/FOXO1/PGC-1*α* signaling pathway, and CG maybe a promising therapeutic candidate for brain injury associated with ischemic stroke. However, more in-depth studies are required to determine the actual role of CG in ischemia stroke.

## Data Availability

The whole uncropped images of the original Western blots, cell morphology pictures, and data of biochemical indicators used to support the findings of this study have been deposited in the 4TU.Centre for Research Data repository 10.4121/uuid:0fceea29-38c8-4f03-a504-1ab324f0524b.

## Abbreviations

Bcl-2: B-cell lymphoma-2
Bax: Bcl-2 associated X protein
CG: Calycosin-7-*O*-*β*-*D*-glucoside
CCK-8: Cell counting kit-8
ELISA: Enzyme-linked immunosorbent assay
FOXO: Forkhead box O
HT22: Hippocampal neuron cell line
LDH: Lactate dehydrogenase
MDA: Malondialdehyde
OGD/R: Oxygen-glucose deprivation/reperfusion
PGC-1*α*: Peroxisome proliferator-activated receptor *γ* coactivator-1
ROS: Reactive oxygen species
RA: Radix Astragali
SOD: Superoxide dismutase
SIRT1: Silent information regulator 1
tPA: Tissue plasminogen activator.
